# Severely Calcified Pericardium Causing Constrictive Pericarditis

**DOI:** 10.14797/mdcvj.1183

**Published:** 2023-01-13

**Authors:** Jagdeep Kaur, Jack Xu, Hani J. Alturkmani, Subhi Al’Aref, Gaurav Dhar

**Affiliations:** 1 University of Arkansas for Medical Sciences, Little Rock, Arkansas, US

**Keywords:** constrictive pericarditis, calcified pericardium, impaired cardiac output

## Abstract

Constrictive pericarditis (CP) is a type of diastolic heart failure caused by an inelastic pericardium that impairs cardiac filling. Diagnosing CP can be challenging, and a variety of imaging techniques may be necessary. We present a unique case of severely calcified pericardium leading to CP.

## Introduction

The pericardium protects the heart and assists in maintaining normal cardiac function.^1,2,3^ Constrictive pericarditis (CP) is a chronic inflammatory process that involves the pericardium. CP can lead to fibrotic thickening, scarring, and calcifications of the pericardium. This can result in abnormal diastolic filling and lead to impaired cardiac output and heart failure.^4,5^ The most common causes of CP include tuberculosis, prior cardiac surgery, chest radiation, and a history of pericarditis.^6,7^ However, many cases are thought to be idiopathic. The most common symptoms are dyspnea on exertion, chest discomfort, edema, and fatigue. On examination of the jugular venous pressure, patients may have a steep, deep y-descent. A pericardial knock may also be auscultated.

Several characteristics are suggestive of CP on echocardiogram. Some of the most sensitive features include a respiration-related ventricular septal shift, pulsus paradoxus, prominent hepatic vein expiratory diastolic flow reversals, inferior vena cava plethora, and a ratio of mitral early (E) to late (A) transmitral filling > 0.8.

In addition to echocardiography, computed tomography (CT) as well as magnetic resonance imaging (MRI) of the chest can better assess the severity of pericardial thickening and calcification.

Some cases of CP can resolve spontaneously or with medical therapy. Medical therapy includes nonsteroidal anti-inflammatory agents, colchicine and corticosteroids. However, in most cases, CP is a chronic and progressive disease. Pericardiectomy is the definitive cure for CP as it usually leads to hemodynamic and symptomatic improvement.^8,9^

## Case Presentation

We describe the case of a 49-year-old female with a history of pericardial calcification, coronary artery disease, and multiple and chronic obstructive pulmonary disease who presented with a several-day history of worsening shortness of breath. On exam, she was noted to have Kussmaul’s sign—impaired jugular vein distension—up to mid-neck, wheezing, and abdominal distention.

**Figure 1:**
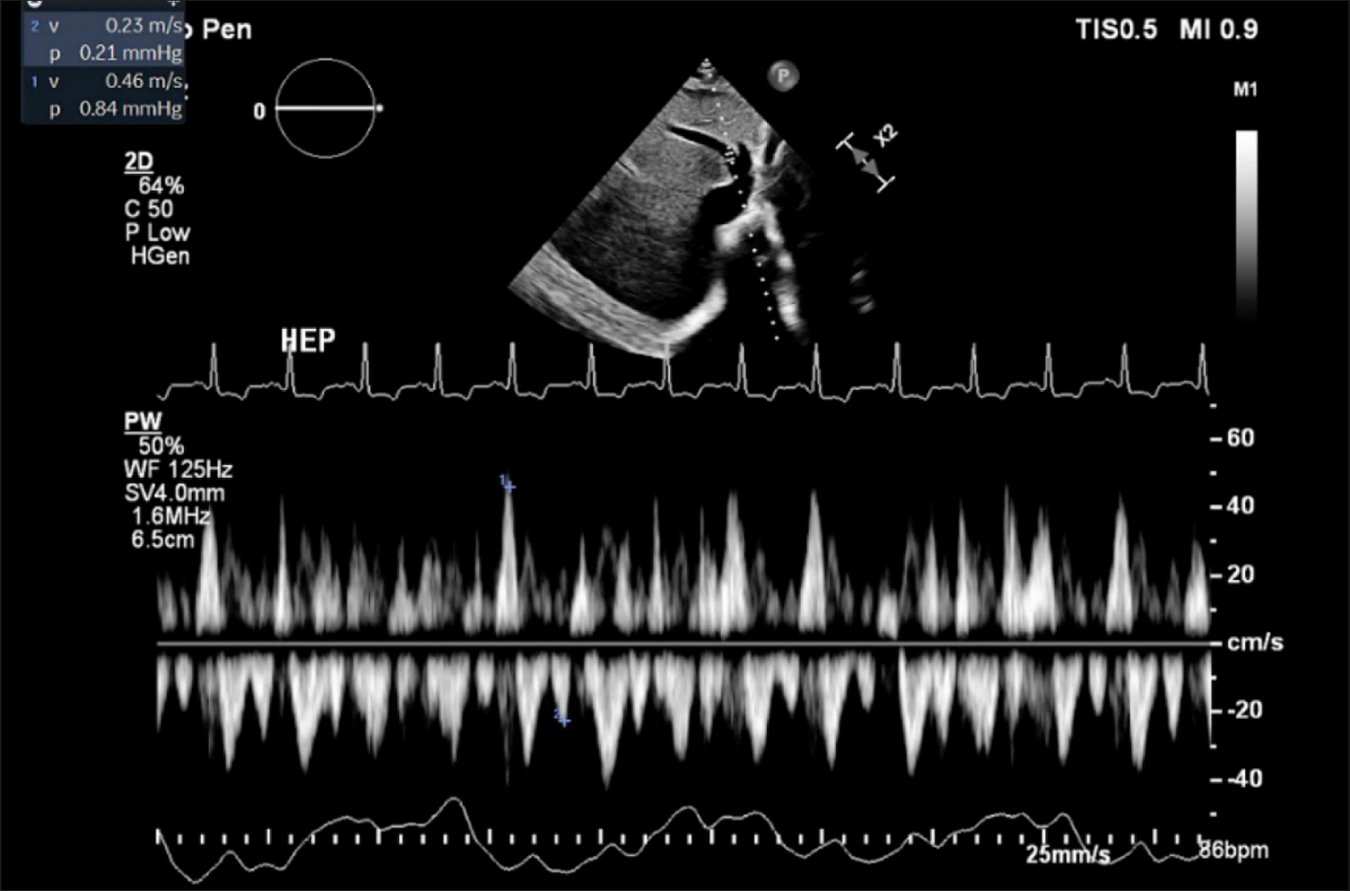
Echocardiogram showing diastolic hepatic vein flow reversal.

**Figure 2:**
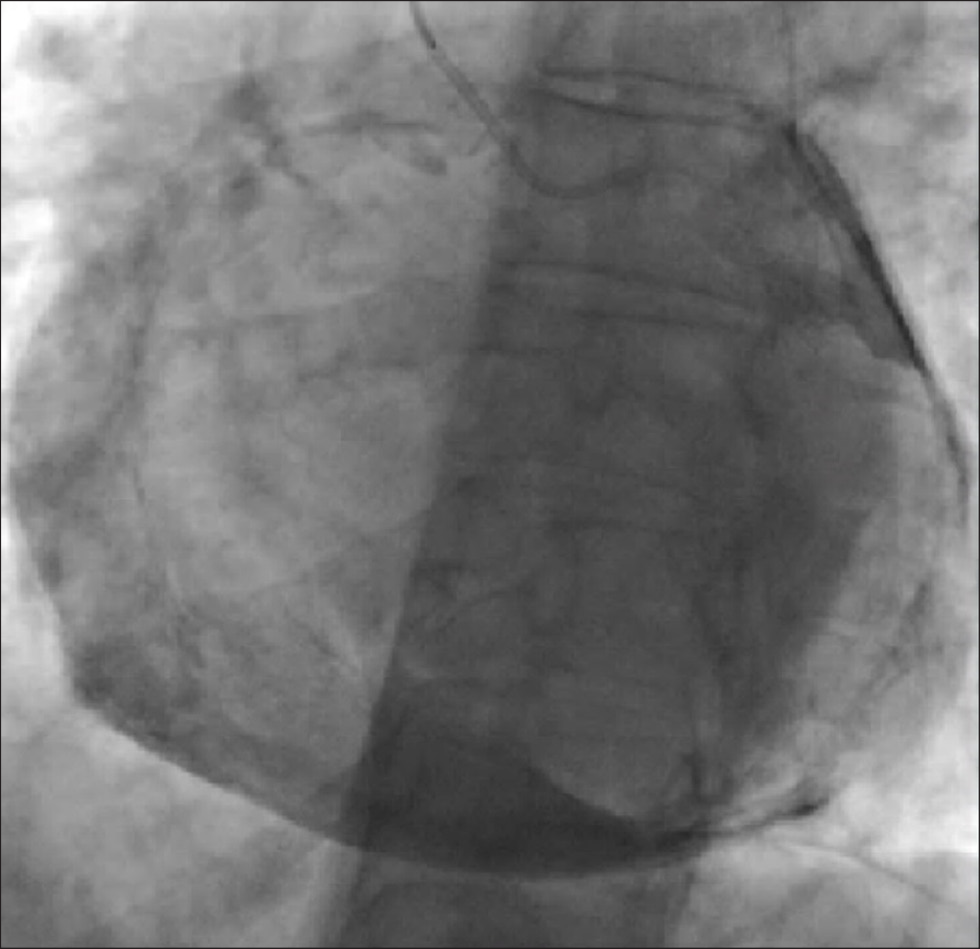
Fluoroscopy showing significant pericardial calcification.

**Figure 3:**
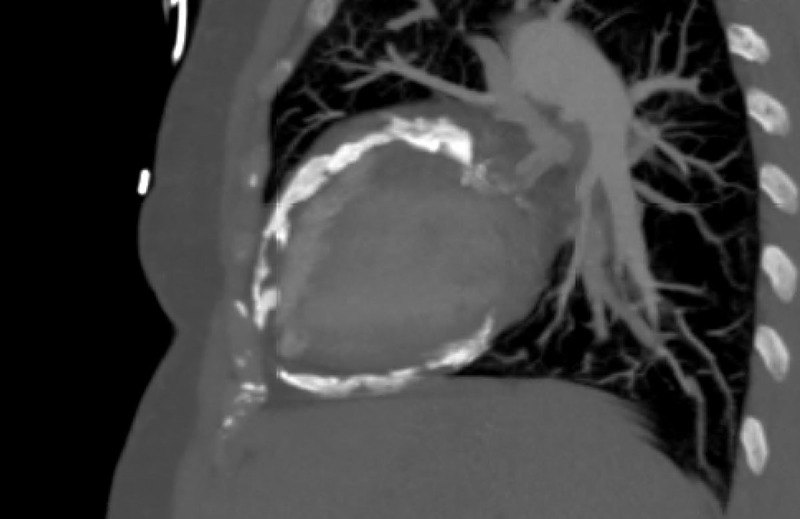
Sagittal view of severe pericardial calcifications on computed tomography.

**Figure 4:**
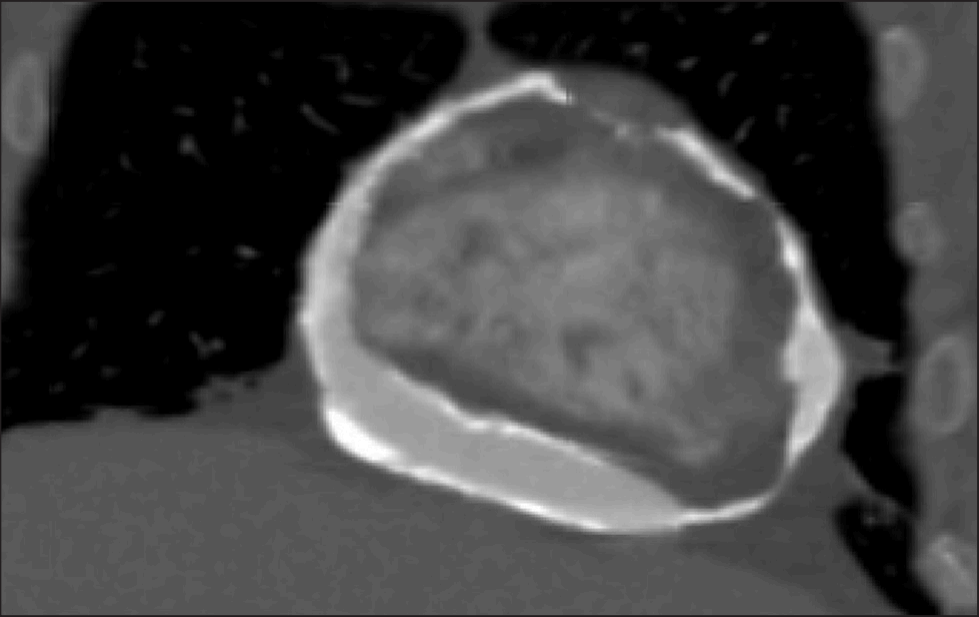
Coronal view of severely calcified pericardium on computed tomography.

On the echocardiogram, she was found to have ventricular interdependence, significant respirophasic variation on the mitral valve, diastolic hepatic vein reversal, and a dilated noncollapsible inferior vena cava (Figure 1). She underwent a coronary angiogram, which showed nonobstructive coronary artery disease but severe pericardial calcifications (Figure 2). A right heart catheterization was not performed due to the echocardiographic findings that were highly suggestive of CP physiology. On CT of the chest, she was found to have severe pericardial calcifications (Figures 3, 4). On cardiac MRI, she was found to have abnormal septal motion of the sigmoidization, septal bounce, and flattening during early diastole. Autoimmune workup (including an antinuclear antibody panel and rheumatoid factor) were negative along with erythrocyte sedimentation rate and C-reactive protein biomarkers. She tested negative for tuberculosis, had no cardiac surgery history, and no history of radiation. She did report a significant history of chest trauma with multiple motor vehicle accidents (with the first being in 2000 and the latest in 2016) and blunt force trauma due to altercations. She had been trialed on nonsteroidal anti-inflammatory drugs (5-day therapy of ketorolac), as well as steroids (5-day therapy of prednisone 40 mg daily), with no relief. She successfully underwent pericardiectomy with resolution of her symptoms. Pericardial biopsy showed fibroconnective tissue with extensive calcifications.

## Discussion

The pericardium is an integral structure for normal cardiac function. In CP, the elasticity of the pericardium is lost due to a chronic inflammatory process, and the pericardium often becomes calcified.^10^ While tuberculosis remains the most common cause of CP in the developing world, the common causes in the developed world are viral, idiopathic, neoplastic, and radiation induced.^11^ However, in this case, with no clear identifiable common cause, the etiology of CP would either be idiopathic or chest trauma.

Though it is thought to be a rare occurrence, the first case of post-traumatic CP is thought to be described by Akenside in 1763.^12^ Since then, though, there are multiple reports of chest trauma being the primary cause of CP. In particular, two case reports highlighted the associated risk of recurrent chest trauma and the development of CP that was related to professional sports, such as boxing and mixed martial arts (MMA).^13,14^ Interestingly, a recent case report from Japan described the development of CP resulting from a back injury after a cyclist was hit by a motor vehicle.^15^

Comparable to the blunt repetitive chest trauma that can be seen with MMA and boxing, our patient had a reported history of chest trauma from both motor vehicle accidents and blunt force trauma during altercations. Echocardiogram showed classic findings consistent with CP (ie, ventricular interdependence), significant respirophasic variation of the mitral valve, and diastolic hepatic vein reversal. Severe pericardial calcifications were incidentally visualized on coronary angiogram, which were later better visualized on CT. Cardiac MRI revealed abnormal septal motion with sigmoidization, septal bounce, and flattening during early diastole. Medical management was attempted initially, but pericardiectomy was ultimately pursued after she had no improvement in her symptoms.

Although pericardial calcifications can be helpful in the diagnosis of CP, echocardiogram findings of respiration-related ventricular septal shift, prominent hepatic vein expiratory diastolic flow reversals, and a ratio of mitral early (E) to late (A) transmitral filling > 0.8 had a sensitivity of 87% and specificity of 91%.^10,16^ Despite the plethora of information one can ascertain from echocardiogram, cardiac MRI has the added advantage of helping distinguish between restrictive cardiomyopathy and constrictive pericarditis. Cardiac MRI findings that have high sensitivity and specificity for constrictive pericarditis include pericardial thickening > 4 mm and septal bounce.^17^

Given the significant history of chest trauma, histopathology not suggestive of infectious disease, and negative autoimmune workup, it was surmised that the most likely cause of this patient’s constrictive pericarditis was trauma.

There have been cases of chest wall trauma leading to pericardial effusion, but very few cases of chest wall trauma have been reported to cause CP.^18^ In conjunction with the rarity of trauma causing CP, the delayed onset of CP from blunt trauma (ranging from 3 to 20 years) adds to the difficulty of diagnosing.^19^ Hence, clinicians should be aware of prior trauma when evaluating a patient for constrictive pericarditis.

## Conclusion

This is a case of severe pericardial calcifications leading to CP. In cases of such extensive pericardial calcification, we believe that patients would be unlikely to benefit from medical therapy. In this patient, given her negative workup for the most common causes of CP, it could be related to her history of repeated chest wall trauma but most likely the etiology is idiopathic.
